# RNA-Seq Analysis for Assessing the Early Response to DSP Toxins in *Mytilus galloprovincialis* Digestive Gland and Gill

**DOI:** 10.3390/toxins10100417

**Published:** 2018-10-16

**Authors:** María Verónica Prego-Faraldo, Luisa Martínez, Josefina Méndez

**Affiliations:** Grupo Xenomar, Departamento de Bioloxía, Facultade de Ciencias and CICA (Centro de Investigacións Científicas Avanzadas), Universidade da Coruña, Campus de A Zapateira, 15071 A Coruña, Spain; veronica.prego@udc.es (M.V.P.-F.); josefina.mendez@udc.es (J.M.)

**Keywords:** DSP toxins, bivalves, mussel, resistance, RNA-Seq, qPCR, metabolism, defense, immunity

## Abstract

The harmful effects of diarrhetic shellfish poisoning (DSP) toxins on mammalian cell lines have been widely assessed. Studies in bivalves suggest that mussels display a resistance to the cytogenotoxic effects of DSP toxins. Further, it seems that the bigger the exposure, the more resistant mussels become. To elucidate the early genetic response of mussels against these toxins, the digestive gland and the gill transcriptomes of *Mytilus galloprovincialis* after *Prorocentrum lima* exposure (100,000 cells/L, 48 h) were de novo assembled based on the sequencing of 8 cDNA libraries obtained using an Illumina HiSeq 2000 platform. The assembly provided 95,702 contigs. A total of 2286 and 4523 differentially expressed transcripts were obtained in the digestive gland and the gill, respectively, indicating tissue-specific transcriptome responses. These transcripts were annotated and functionally enriched, showing 44 and 60 significant Pfam families in the digestive gland and the gill, respectively. Quantitative PCR (qPCR) was performed to validate the differential expression patterns of several genes related to lipid and carbohydrate metabolism, energy production, genome integrity and defense, suggesting their participation in the protective mechanism. This work provides knowledge of the early response against DSP toxins in the mussel *M. galloprovinciali*s and useful information for further research on the molecular mechanisms of the bivalve resistance to these toxins.

## 1. Introduction

Nowadays, harmful algal blooms (HABs) constitute one of the most important sources of natural contamination in the marine environment. This term refers not only to the phenomena originated by the proliferation of harmful algae, but also the phenomena caused by proliferation of toxic algae [1]. Although there is still a considerable absence of high quality time-series data in most regions affected by HABs [2], the blooms caused by the outbreaks of diarrhetic shellfish poisoning (DSP) toxin producing species seem to be associated with most of the HABs detected in European coasts [3]. These toxins are produced by dinoflagellates of the *Dinophysis* and *Prorocentrum* genera and constitute a heterogenous group of polyethers, including okadaic acid (OA) and its analogs, the dinophysis toxins (DTXs) [3,4,5,6,7,8]. In terms of abundance and consequent toxicity, OA is considered the main DSP toxin followed by DTX1, while DTX3—a less abundant DSP toxin—has become important because of its production through metabolic transformations that occur in some bivalves [7]. DTX1 seems to have similar toxicity levels to that of OA, while DTX2, DTX3 and DTX4 are less acutely toxic. On the other hand, the acylation of the 7-hydroxyl group with a saturated fatty acid forms compounds which are approximately 20 times less toxic than OA [9]. DSP toxins have a high lipophilic character, which allows for them to be accumulated in the fatty tissues of filter-feeding organisms—mainly in bivalve mollusks—and be transferred across the food chain, causing several gastrointestinal disorders [6]. Currently, efficient monitoring programs have been established by many countries to ban the harvesting of contaminated seafood and therefore, avert human intoxications [3]. However, seafood with small quantities of DSP toxins is still commercialized.

Since the ability of OA to inhibit several types of serine/threonine protein phosphatases was discovered by Bialojan and Takai [4], numerous works have studied the harmful effects of this toxic compound on different model systems, including different mammalian cell lines [8]. However, studies that assess the effects of these toxins in their main vectors—bivalve mollusks—are scarce. Recent studies carried out by our research group showed that DSP toxins cause more severe genotoxic and cytotoxic effects in bivalve cells at low concentrations and short exposition times, while these effects decrease or disappear as exposure increases in concentration and time [5,10,11,12]. This suggests that these organisms may have developed a quick protection mechanism against these toxic compounds. This may be associated with the accumulation, transformation and elimination of DSP toxins. This still unknown mechanism is of great interest for predicting the time course of toxic episodes and for reducing their negative consequences. With the aim of obtaining knowledge about this early genetic response, our research group has assessed the immediate effects caused by DSP toxins in the mussel *Mytilus galloprovincialis* using different stress indicators: DNA breaks, number of apoptotic cells [12], lipid peroxidation and antioxidant enzyme activities [10]. Although these indicators constitute a good approach to assess the first harmful effects produced by these toxins, they offer just a partial view on mussel response to toxic compounds. Taking this into account, it seems necessary to carry out analyses on the transcriptome response of mussels to DSP toxins to obtain a global perspective on their defense mechanisms against these toxins. Previous works used transcriptomic techniques to determine *M. galloprovincialis* transcriptome response to several stimuli, including marine toxins and pathogens [13,14,15,16,17,18,19]. Transcriptomic techniques such as RNA-Seq provide a valuable contribution to determining which gene pool expression is induced or suppressed depending on its physiological role in response to different treatments [20].

Some works have determined that the accumulation and distribution of DSP toxins in mussels is tissue specific [21,22]. The digestive gland is the mussel tissue that accumulates the most DSP toxins and is considered the main site of toxin bioconversion [23]. Furthermore, gills have numerous functions related to feeding, digestion and elimination of wastes and contaminants. The large surface and thin epithelium of the mussel gill make it an efficient site for direct interaction with the environment. Thus, gills efficiently capture suspended food particles—thanks to the mucus produced by them—and mediate their transport through the mussel mouth and digestive system [24].

In this work the whole transcriptome of the mussel *M. galloprovincialis* was de novo assembled and differentially expressed genes (DEGs) in digestive gland and gill after early exposure to DSP toxin-producer *Prorocentrum lima* were identified in order to determine the first response of these bivalve mollusks to these toxins and identify transcripts which could participate in the resistance mechanisms of mussels against the harmful effects of DSP toxins. Previous studies have characterized gene expression changes related to exposition to OA in bivalve mollusks [17,18,25,26] but to our knowledge, this is the first work that uses RNA-Seq to study the early transcriptional response of the mussel *M. galloprovincialis* to DSP toxins under short exposure to low concentrations of *P. lima*.

## 2. Results

### 2.1. Toxin Accumulation

According to the High Performance Liquid Chromatography/Mass Spectrometry (HPLC/MS) analyses, the *P. lima* strain AND-A0605 had an average toxin content of 0.4 pg OA/cell. Control mussels, fed with a mixture of *Isochrysis galbana* and *Tetraselmis suecica*, did not accumulate OA (<0.1 ng/g dry weight), while OA accumulated in treated mussels—fed also with *P. lima*—was 112.12 ng/g dry weight. Based on these results, and since these levels are well below the limit allowed by the European Commission Regulation for harvesting and sale (160 µg of OA equivalent/kg dry weight), we could consider that the mussels were exposed to low microalga cell densities, similar to those at the early stages of a HAB [27].

### 2.2. Transcriptome Sequencing and De Novo Assembly

In order to investigate the defense mechanisms of mussels exposed to DSP toxins, eight libraries derived from the digestive gland and the gill of the mussel *M. galloprovincialis,* in the absence of and under low densities of *P. lima* exposure, were constructed and sequenced using an Illumina sequencing platform. After de novo assembly with Trinity and Oases and their subsequent clustering by homology, 95,702 transcripts were obtained. Mean transcript size was 748 bp, with lengths ranging from as small as 100 bp to as a large as 16,082 bp. About 78% of the final assemblies were >200 bp and a N50 length of 1062 bp was obtained (Table 1).

### 2.3. DEGs Among Samples

**Figure 1:**
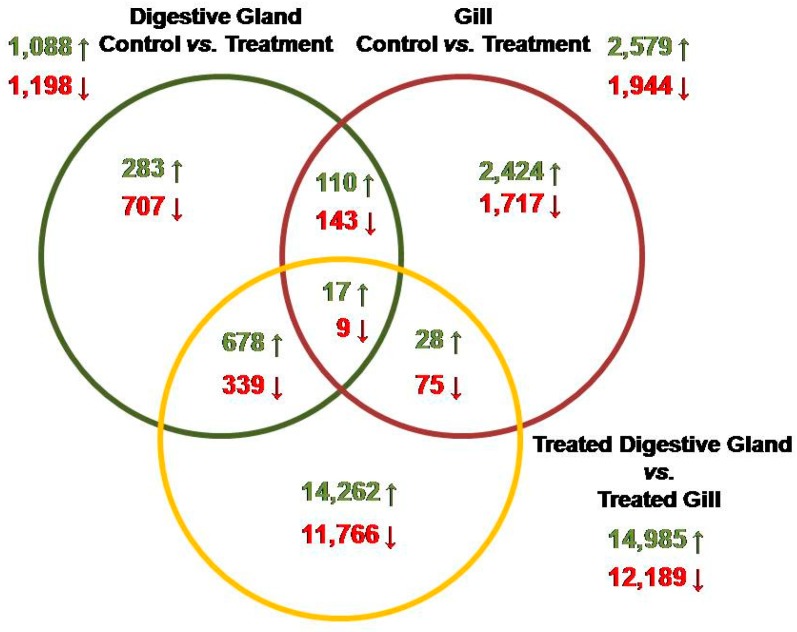
Venn diagram indicating the overlaping of genes significantly up-regulated (green arrows) and down-regulated (red arrows) when DEGs from different libraries were compared.

Transcriptomic analyses were performed with the aim of identifying the main molecular mechanisms involved in the response of mussels to early contamination by DSP toxins. Using a RNA-Seq experiment, we generated transcriptome profiles for the digestive gland and the gill of the mussel *M. galloprovincialis* exposed to low densities of *P. lima* (100,000 cells/L) for a short period of time (48 h) and compared these data with profiles obtained from the digestive glands and the gills of control mussels. Sequences of all DEGs obtained are listed in File S1. A Venn diagram was used to depict the overlapping of DEGs when libraries were compared (Figure 1). Regarding the digestive gland, there were a total of 2286 DEGs between treatment and control groups, from which 1198 and 1088 transcripts were up- and down-regulated, respectively. Regarding the gills, there were a total of 4523 DEGs between both groups (treatment and control), from which 2579 and 1944 transcripts were up- and down-regulated, respectively. As a complementary analysis, the comparison of treated digestive glands and gills showed a total of 27,174 DEGs; 14,985 of them were up-regulated transcripts, while 12,189 were down-regulated (File S2). Only 26 transcripts out of all DEGs obtained were detected in all comparisons, with 17 and 9 of them being up- and down-regulated, respectively. The comparison of digestive glands and gills showed a total of 253 DEGs, from which 110 and 143 transcripts were up- and down-regulated, respectively. These DEGs could be useful for discovering genes involved in the early response to DSP toxins and, thereby, for identifying putative biomarkers for monitoring in advance of contamination episodes in the marine environment.

### 2.4. Gene Functional Annotations

Only 6% of the contigs included in the reference transcriptome showed BLAST similarity to proteins. About 20% of transcripts showed similarity to protein sequences deposited in the UniProt database and approximately 50% showed Pfam annotations. Thus, a relevant fraction of the contigs included in the reference transcriptome obtained in this work did not display any BLAST similarity or annotation.

Table 2, Table 3, Table 4 and Table 5 show the 25 most significantly up- and down-regulated genes in the digestive gland and the gill after exposure to low concentrations of DSP toxins (100,000 cells/L) for a short time period (48 h). Among the top over-represented DEGs in the digestive gland are genes that encode enzymes involved in the electron transport chain or mitochondrial oxidative phosphorilation (cytochrome c oxidase), as well as genes that encode ribosomal proteins or proteolytic enzymes (ribosomal protein L23a) (Table 2). Among the infra-represented genes in this tissue are also genes that encode enzymes of the electron transport chain (NADH dehydrogenase subunit 5) and ribosomal proteins (40S ribosomal protein S10-like). On the other hand, there are genes related to apoptosis (GTPase IMAP family member 7) and genes that encode proteins involved in the formation of nacre, promoting the crystallization of calcium carbonate (Perlucin) (Table 3). Similar to the digestive gland, among the over-represented genes in the gill (Table 4) are genes that encode enzymes of the electron transport chain (NADH dehydrogenase subunit 6) and proteins that play a role in the regulation of ion transport (calcyphosin-like protein). In contrast to the results obtained in the digestive gland, a gene encoding the cytochrome c oxidase subunit I is significantly down-regulated (Table 5). Also, a gene that encodes a protein involved in lipid metabolic processes and endocytosis is down-regulated in this tissue in the early response to DSP toxins (Table 5).

Functional enrichment studies performed using Pfam annotations obtained from the DEGs, showed 44 and 60 Pfam families significantly enriched in the digestive gland and the gill, respectively (File S3). Among these enriched domains, we found genes coding for proteins involved in GTP and calcium ion binding, transport, antibacterial activity and immune system in the digestive gland (Table 6). On the other hand, domains related to cell adhesion, cell-cell recognition, protein binding, immune system and correct folding of proteins were found in the gill (Table 7).

**Figure 2:**
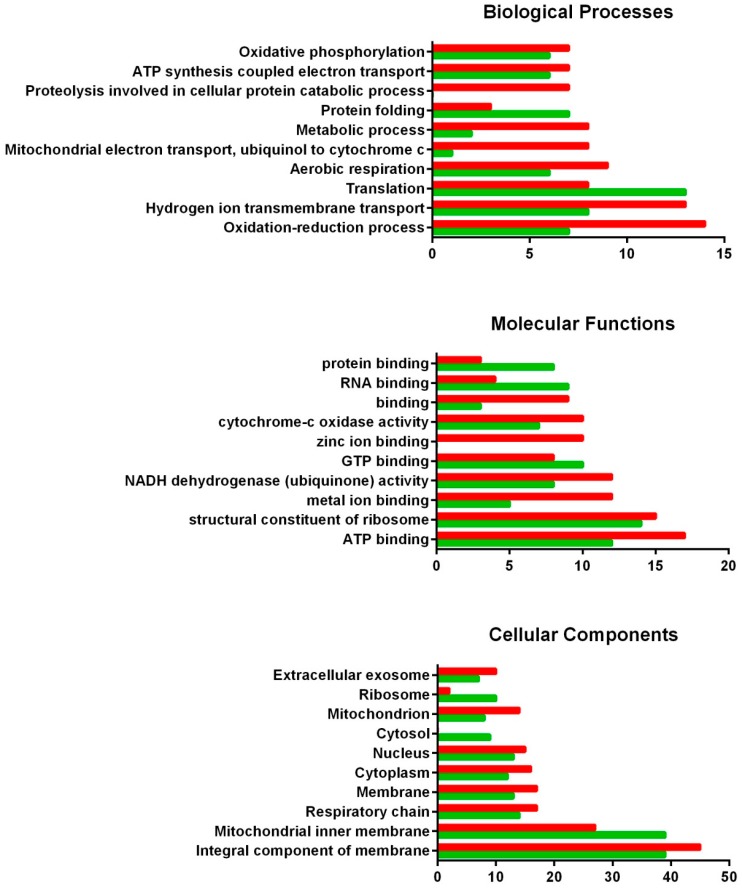
GO classification of DEGs from the digestive gland of the mussel *M. galloprovincialis* exposed to the DSP toxin-producing dinoflagellate *P. lima*. Overrepresented and infrarrepresented biological processes, molecular functions and cellular components are shown. Red and green bars represent the number of down- and up-regulated genes in each category, respectively. The length of the bars is determined by the number of genes identified within each subcategory.

**Figure 3:**
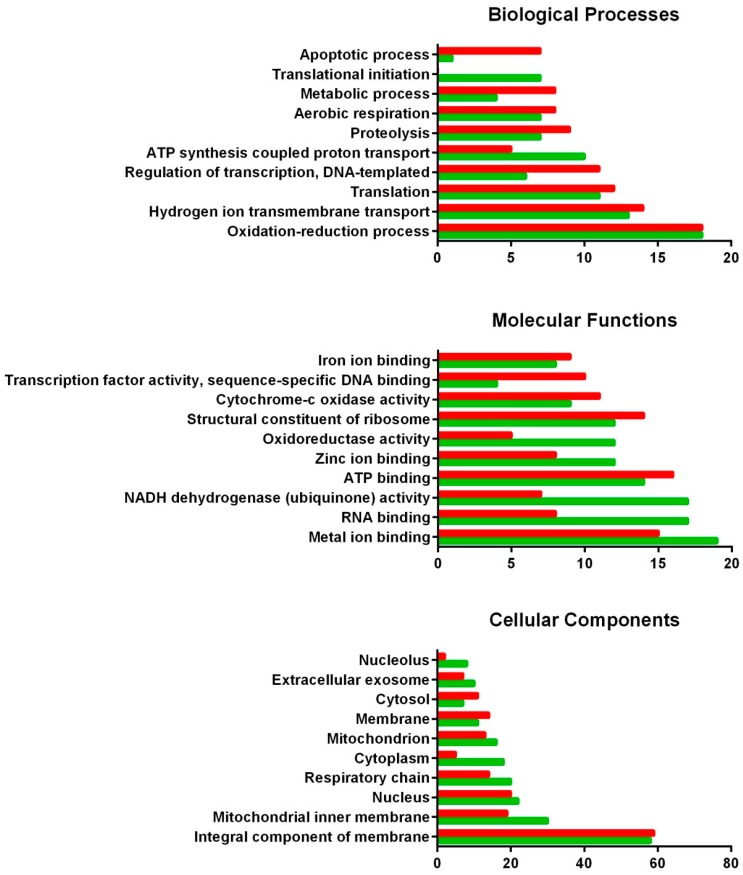
GO classification of DEGs from the gill of the mussel *M. galloprovincialis* exposed to the DSP toxin-producing dinoflagellate *P. lima*. The overrepresented and infrarrepresented biogical processes, molecular functions and cellular components are shown. Red and green bars represent the number of down- and up-regulated genes in each category, respectively. The length of the bars is determined by the number of genes identified within each subcategory.

All DEGs from each tissue were classified according to the three main Gene Ontology (GO) aspects (biological processes, molecular functions and cellular components) and subcategories within (Figure 2 and Figure 3). Among the biological processes obtained for the digestive gland, proteolysis involved in the cellular protein catabolic process deserved special recognition for its down-regulation, while protein folding and translation are two of the most up-regulated processes. Regarding molecular functions, zinc and metal ion binding, as well as NADH dehydrogenase activity, showed considerable down-regulation in the digestive gland exposed to DSP toxins, while protein, GTP and RNA binding were up-regulated when the digestive gland responded to these toxins. The cellular components most involved in the response against DSP toxins seem to be the cytosol and the mitochondrion (cellular components up-regulated), while numerous sequences related to the extracellular exosome are down-regulated.

Regarding gills, the main down-regulated biological process when this tissue is exposed to DSP toxins is apoptosis. On the contrary, processes such as translational initiation or ATP synthesis coupled proton transport are over-represented after exposure to DSP toxins. When molecular functions are considered, RNA binding and NADH dehydrogenase activity are mostly up-regulated, while iron ion binding, sequence-specific DNA binding or cytochrome c oxidase activity are mainly down-regulated in the presence of DSP toxins. In this tissue, those cellular components most involved in the response against DSP toxins seem to be the nucleolus and the mitochondrion.

### 2.5. Real-Time Quantitative PCR (qPCR) Validation

We selected 10 DEGs for real-time qPCR confirmation based on their functions (lipid metabolism and immunity): seven up-regulated, two down-regulated and one with no differential expression. Regarding the digestive gland, big defensin 2 (BD2), NADH dehidrogenase subunit 5 (NADH5) and KAZAL domain containing protein (KAZAL DC) were up-regulated, GIY-YIG domain containing protein (GIY-YIG DC) was down-regulated and Dynactin-subunit-6-like (DYNA) showed no expression changes. Regarding the gills, Cytosolic phospholipase A-2 like (CPLA2), Arachidonate 15-lipoxygenase B-like (ALOX15B), Alpha-L-fucosidase-like (FUCA) and H_Lectin domain containing protein (H_Lectin DC) were up-regulated, while Fibrinogen_C domain containing protein (Fibrinogen_C DC) was down-regulated.

**Figure 4:**
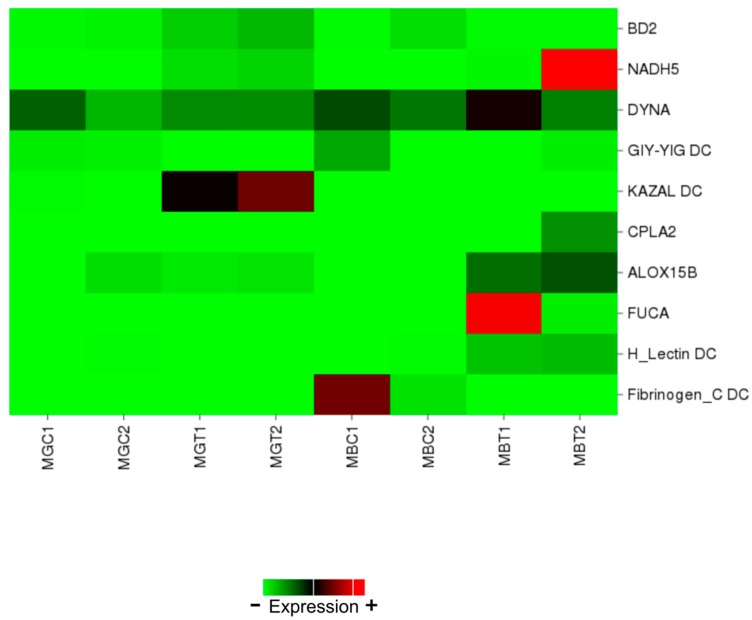
Heatmap showing expression levels of a set of annotated genes involved in the early response to DSP toxins in mussels and selected for qPCR validation. Columns represent one library each and cells depict gene expression levels based on the number of reads. MGC: library obtained from digestive glands of control mussels. MGT: library obtained from digestive glands of treated mussels. MBC: library obtained from gills of control mussels. MBT: library obtained from gills of treated mussels.

The heatmap provided in Figure 4 illustrates the expression levels of these genes in each library.

To confirm these patterns of expression by means of real-time qPCR, specific primers were designed. Sequences of these primers are shown in Table 8.

NormFinder software showed that rpS4 and TPM genes were the most stable genes and identified them as the best two-gene combination among all potential reference genes. These two genes also showed the lowest SD values when analyzed with BestKeeper. Moreover, their suitability as reference genes was supported by RefFinder results. Therefore, taking into account the combination of all results from the different analysis methods used (Table 9), TPM and rpS4 were identified as the most stable pair of reference genes in the digestive gland. These two genes were used for the normalization of gene expression in real-time qPCR.

**Figure 5:**
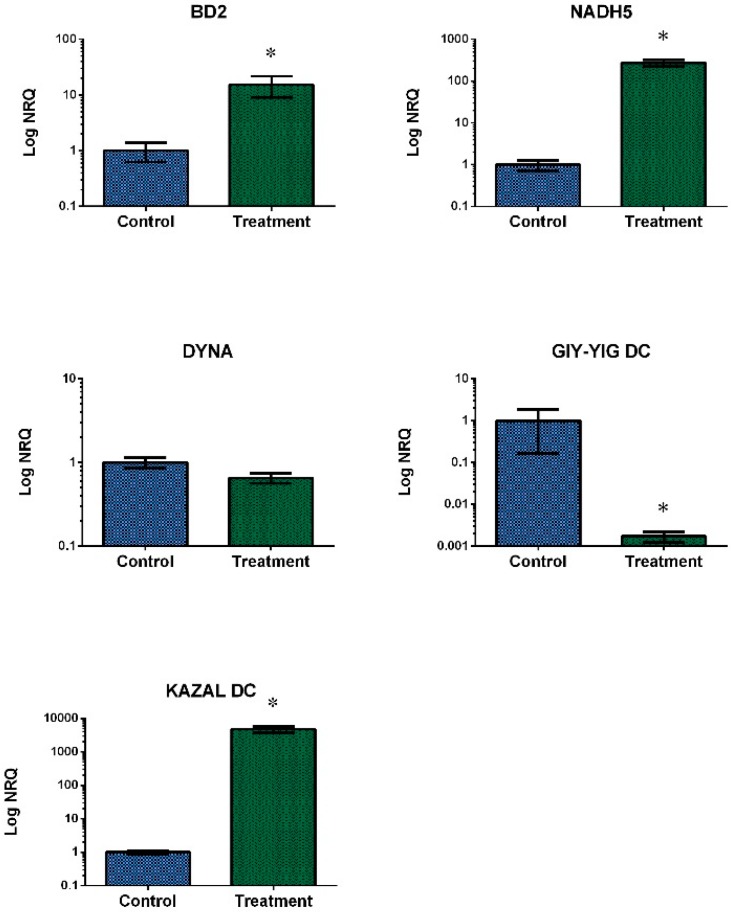
Relative transcript levels for each selected gene of digestive gland of the mussel *M. galloprovincialis* exposed to the DSP toxin-producing dinoflagellate *P. lima*. Blue bars: control samples. Green bars: samples treated with 100,000 cells/L for 48 h (mean ± SE). NRQ: Normalized Relative Quantification. *n* = 4. * indicates significant differences to control according to Mann-Whitney’s U-test (*p*-value < 0.05).

**Figure 6:**
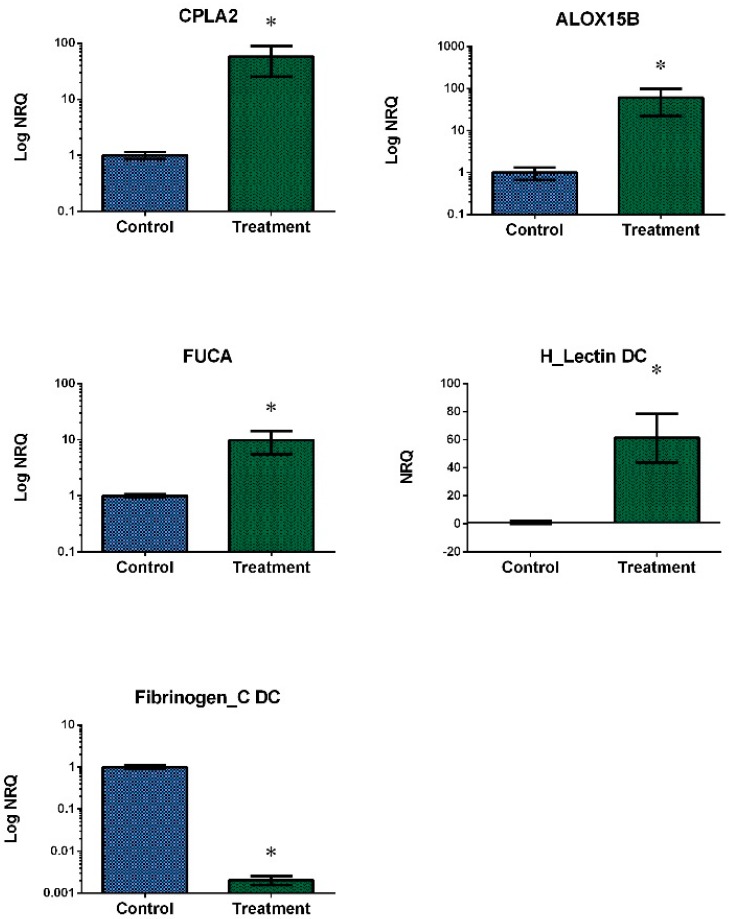
Relative transcript levels for each validated candidate gene of gill of the mussel *M. galloprovincialis* exposed to the DSP toxin-producing dinoflagellate *P. lima*. Blue bars: control samples. Green bars: samples treated with 100,000 cells/L for 48 h (mean ± SE). NRQ: Normalized Relative Quantification. *n* = 4. * indicates significant differences to control in Mann-Whitney’s U-test (*p*-value < 0.05).

The results of normalized expression (Figure 5 and Figure 6) validated the previous observations obtained using RNA-Seq. Fibrinogen_C DC, FUCA and NADH5 qPCR analyses were carried out using three biological replicates.

## 3. Discussion

Given the scarce knowledge of the resistance mechanisms involved in the early response of bivalve mollusks to marine toxins, the data presented in this work represent an important resource. Compared to other transcriptional works carried out in the digestive gland of the mussel *M. galloprovincialis* [13,15], a great number of DEGs were identified in the present study. This suggests a major impact of DSP toxins on gene expression regulation in the digestive gland and the gill of this species.

This study also revealed numerous transcripts assigned to Pfam families related to transport, cell adhesion, protein binding, calcium-binding proteins or immune system, among others. Many of these domains were also identified when haemolymph and digestive gland transcriptomes of mussel were analyzed in response to *Vibrio alginnolyticus* infection and domoic acid exposure [15,28,29]. Previous works carried out in bivalves exposed to marine toxins have shown significant changes in the expression levels of genes and proteins related to detoxification processes, such as cytochromes p450, ATP-binding cassette (ABC) transporters or glutathione S-transferases (GST) [12,15,30,31]. Surprisingly, although some of these genes are included among the DEGs in our results, they were not found among the most significant ones. Guo et al. [32] suggested the possible implication of p450 genes in OA metabolism in humans, generating new metabolites with less capacity to inhibit PP2A in comparison to OA. However, these transformations would not be completely effective to OA detoxification, which could explain our results.

Regarding GO, cellular organization and biogenesis, protein metabolism and modification, catabolism, response to stress and death and cell death are some of the biological processes most involved in the mussel response against toxins [33]. This is partially consistent with the main biological processes assigned in the present work when the digestive gland and the gill of mussels were exposed to DSP toxins. However, our data showed an important down-regulation of genes related to metabolic and apoptotic processes in the digestive gland and the gill, respectively, which may lay behind the first harmful effects of DSP toxins in these tissues. This result is not in agreement with the apoptosis induction observed in digestive glands when Mediterranean mussels were fed OA-contaminated nutrients [19]. Among the molecular functions involved in mussel response to toxins are protein binding, catalytic activity and transporter activity [33]. A similar result was obtained in the present work, although with important cytochrome-c oxidase and NADH dehydrogenase activities. On the other hand, the main cellular components shown in comparative transcriptomic studies of bivalves exposed to toxins were cytoplasm, nucleus, extracellular region and mitochondrion [33]. This is in agreement with some of the cellular components identified in the present work. However, our results also seem to show a key role of the extracellular exosome and respiratory chain in both mussel tissues—the digestive gland and the gill—in the early response to DSP toxins. Yamashita et al. [34] had already determined that exosomal secretion mechanisms are essential for methylmercury detoxification in the zebrafish embryo. Also, our work revealed an important participation of membrane integral components in the response to DSP toxins. This may be related to the known inhibitory effect of OA on intercellular channels in mammalian cells [35].

A large amount of contigs included in the reference transcriptome obtained in this study did not display any BLAST similarity or annotation, even with the recently sequenced *M. galloprovincialis* genome [36] or with *Crassostrea gigas* genome [37]. That was also the case for many of the top DEGs identified in this work that, despite their implication in the early response of mussel to DSP toxins, could not be identified. Similar results were obtained in a previous RNA-Seq study when digestive gland transcriptome of *M. galloprovincialis* was analyzed after exposure to the dinoflagellate *Alexandrium minutum*, a paralytic toxin producer [13]. Taking into account the length of some of these contigs as well as previous suggestions made by some authors, these sequences could be candidates to long non-coding RNA (lncRNA). lncRNA can regulate the activity of other genes by interacting with protein-coding mRNAs [38]. Milan et al. [39] observed that approximately 10% of the contigs obtained from the transcriptome of the clam *Ruditapes philippinarum* were originated by natural antisense transcription (NAT), a process that seems to be highly prevalent in bivalves.

When the data represented in the heatmap and the results obtained by qPCR were compared, a high correlation was observed between them, clear evidence that the RNA-Seq analysis conducted in this work was robust. Analyses in the digestive gland showed that the two most suitable genes for qPCR gene expression normalization were rpS4 and TPM. This result is in agreement with previous reports in which rpS4 was proposed as an optimal housekeeping gene to use under similar conditions [10,40]. To our knowledge, this is the first time that TPM is proposed and used in mussels to normalize qPCR data.

Our digestive gland data showed the up-regulation of different genes related to immune defense, including BD2, NADH5 and a KAZAL DC protein. Big defensins belong to a diverse family of peptides not only in terms of sequence, but also in terms of genomic organization and regulation of gene expression [41]. High gene expression levels of big defensin were identified in gills of the mussel *Bathymodiolus azoricus* [42]. Also, their up-regulation in haemocytes of the oyster *C. gigas* exposed to *A. minutum*—a paralytic toxin producer—has been associated with alterations in the immune system [43]. Similarly, big defensin gene expression was significantly up-regulated in haemolymph of the scallop *Argopecten irradians* when it was exposed to OA [44]. However, in a work in which the mussel *M. galloprovincialis* was exposed to the marine contaminant tris (1-choro-2-propyl) phosphate (TCPP) big defensin was down-regulated, affecting immunocompetence. Taking into account the high diversity of these genes [41] the big defensin identified in our work may correspond to a new variant of *M. galloprovincialis* related to the response to DSP toxins. On the other hand, the NADH protein family participates in transport and energy production. NADH is the third most frequently detected protein in comparative transcriptional studies that are carried out in bivalves exposed to different toxins [33]. Our results showed a significant increase in NADH5 gene expression. This is in line with an important up-regulation of NADH observed in a microarray designed based on data from normalized and suppression hybridization (SSH) libraries obtained from digestive gland and gill of the mussel *M. galloprovincialis* after exposition to sublethal concentrations of OA [17]. Our results also showed high expression levels of a putative KAZAL DC protein. Gerdol and Venier [45] have suggested that some bivalves can express Kazal-like protease inhibitors to counteract protease variants produced by invading microbes.

On the contrary, our digestive gland data showed the down-regulation of a putative GIY-YIG DC protein. This domain is present in many endonucleases involved in cellular processes such as DNA repair, the restriction of incoming foreing DNA, the movement of non-LTR retrotransposons or the maintenance of genome stability [46]. Indeed, Biscotti et al. [47] suggested that the expansion of this family in lungfish might be a genomic defense mechanism against the threat of spreading mobilome. Furthermore, Dittrich et al. [48] reported a gene which contains a GIY-YIG nuclease domain as an essential gene for proper DNA damage response in *Caenorhabditis elegans* embryos. However, mutants for this gene seem to have normal cell cycle arrest and apoptosis, which means this gene is not involved in the initial signalling process following DNA damage. This fact might partially explain the down-regulation of this transcript in the digestive gland of mussels during the early stages of DSP exposure, the situation simulated in the present study.

Our gill data showed an up-regulation of different genes related to lipid and carbohydrate metabolism, inflammatory response or immune defense, including CPLA2, ALOX15B, FUCA and a H_Lectin DC protein. CPLA2 is an enzyme that plays an important role as the primary generator of free arachidonic acid (AA)—a common precursor of a family of compounds with roles in inflammation [49]—released from membrane phospholipids. CPLA2 expression and activity are increased by reactive oxygen species (ROS) [50]. However, in a previous work, a decrease in lipid peroxidation levels was observed when mussel gills were exposed to the same DSP treatment [10]. This suggests the existence of an alternative defense mechanism. On the other hand, lipoxygenases (LOX) catalyze the generation of leukotrienes from AA producing byproducts that can function as ROS [51]. Some mussel extracts contain fatty acids with the ability to inhibit AA oxygenation by the cycloxigenase and LOX pathways, thus preventing inflammation [52]. In mammals, CPLA2 can cause membrane degradation, changes in plasma and mitochondrial membrane bioenergetics and permeability [53] and lysosomal membrane destabilization [54]. Indeed, CPLA2 is used as a stress indicator in biomonitoring programs. Some authors have also suggested that the up-regulation of genes involved in the inflammatory process, which was observed when digestive glands of the oyster *C. gigas* were exposed to *P. lima*, might represent a risk to this bivalve’s integrity [55]. Heavy metals functionally alter lysosomal membranes in haemocytes of mussels [56]. Ca^2+^ dependent CPLA2 enzymes play an important role in the lysosomal membrane destabilization induced by mercury and copper in the haemolymph cells of mussels [57]. Mussel gill exposed to low DSP toxin concentration produces an inflammatory response associated with the up-regulation of CPLA2 and ALOX15B that may be partially compensated by the up-regulation of antioxidant enzymes shown in many studies [10,58].

FUCA is an enzyme located in lysosomes and involved in carbohydrate metabolism. Based on our results, this gene seems to take part in the early response of mussel gills to DSP toxins. However, FUCA did not show gene deregulation when the gill of the scallop *Nodipecten subnodosus* was exposed to *Gymnodinium catenatum*, while an up-regulation was observed in the adductor muscle [59]. A down-regulation of FUCA protein was observed when the scallop *Pecten maximus* was exposed to hypoxia at different temperatures, suggesting an energy saving strategy by reducing protein turnover [60]. Nevertheless, the restriction of carbohydrate metabolism does not seem to be an important part of the early response of mussel gill to DSP toxins. Our gill data also showed up-regulation of a putative H_Lectin DC protein. This is a common finding in this type of studies, since type C lectins are usually overrepresented in bivalve transcriptomes exposed to marine toxins [17,61]. However, there is still relatively little information available about this domain related to cell adhesion and carbohydrate binding.

On the other hand, our gill data showed the down-regulation of a putative Fibrinogen_C DC protein. A study about the immune system of the mussel *M. galloprovincialis* identified fibrinogen as one of the most abundant transcripts in the Mytibase collection [62]. More specifically, C-terminal fibrinogen-like domain has a structure that binds to the carbohydrate residues of foreing and apoptotic cells. Indeed, some fibrinogen-like domains are included in many lectins [63] and, consequently, are involved in microorganism recognition by the activation of the lectin pathway, constituting a first line of immune defense. Although fibrinogen was first associated with haemolymph, the gill together with the digestive gland were the following tissues with the highest gene expression levels when three fibrinogen-related proteins were evaluated in the mussel *M. galloprovincialis* [64]. Down-regulation of fibrinogen was also observed when haemolymph of the scallop *A. irradians* was exposed to low concentrations of OA (50 nM) for short exposure times (48 h), suggesting the potential of this toxin to inhibit the ability of scallops to recognize and remove non-self particles [65]. Gene expression levels of Fibrinogen C also decreased when bay scallop gill tissue was exposed to 500 nM of OA for 48 h [30]. Differences in gene expression of fibrinogen C were also detected in the digestive gland of the mussel *M. galloprovincialis* after exposure to domoic acid-producing *Pseudo-nitzschia* [15]. However, fibrinogen gene expression was significantly up-regulated when the haemolymph of the scallop *A. irradians* was challenged with *Listonella anguillarum* [66] or when the haemolymph of the mussel *Mytilus chilensis* was exposed to saxitoxins [58]. It is important to note that, as in the case of big defensins, proteins that contain this domain present high individual variability. Thus, different mussels usually have different gene sequences, which demonstrates the extraordinary complexity of the immune system in these organisms [62].

## 4. Conclusions

This work represents the first RNA-Seq approach used in the mussel *M. galloprovincialis* to analyze tissue-specific mussel transcriptome after early exposure to DSP toxins. It describes the transcriptome and gene expression profiles of *M. galloprovincialis* digestive gland and gill, therefore increasing available genomic resources for this organism.

Furthermore, results showed that DEGs in early response to DSP toxins include genes involved in defense, immunity and metabolism, sheding some light into the resistance mechanisms that these organisms have against harmful effects of DSP toxins. In the digestive gland, BD2, KAZAL DC and NADH5 genes were up-regulated while GIY-YIG DC was down-regulated and DYNA showed no expression changes. On the other hand, ALOX15B, H_Lectin DC, CPLA2 and FUCA genes were up-regulated and Fibrinogen_C DC was down-regulated in gill. Nevertheless, many of the genes that responded to these toxins have been described as DEGs in response to other stimuli, indicating that the mussel defense reaction is to some extent unspecific, which may be beneficial when faced with other potentially harmful compounds.

This study also indicated that the expression of rpS4 and TPM genes in the digestive gland under these experimental conditions is stable and, therefore, these genes can be employed as reference genes to normalize gene expression in qPCR experiments carried out in mussels exposed to low concentrations of DSP toxins for short time periods.

## 5. Materials and Methods

### 5.1. Sample Collection and Experimental Design

**Figure 7:**
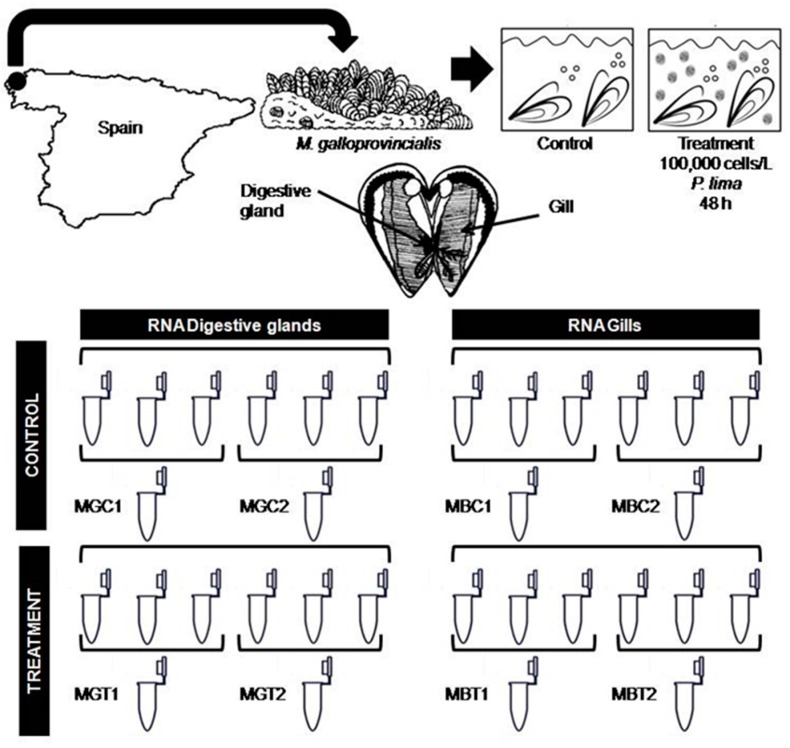
Experimental design diagram. Mussels from rocky shores were acclimated to laboratory conditions and subsequently exposed to 100,000 cells/L of *P. lima* for 48 h. Afterwards, gills and digestive gland were used for RNA extraction. RNA from 3 individuals was pooled for library construction and sequencing. MGC: RNA pool obtained from digestive glands of control mussels. MGT: RNA pool obtained from digestive glands of treated mussels. MBC: RNA pool obtained from gills of control mussels. MBT: RNA pool obtained from gills of treated mussels.

Adult individuals of the mussel *M. galloprovincialis* (34 ± 0.5 mm anterior-posterior shell length) were collected from a natural population in the rocky shores of O Rañal beach (43°19′40.1″ N, 8°30′45.1″ W, A Coruña, NW Spain) in April 2015. This location (used by our research group in other studies [10]) was chosen as our sampling site based on its low density of DSP toxin-producing dinoflagellates [67]. The invertebrate animal experiment was assessed by the Spanish Ministry of Economy and Competitivity (project AGL2012-30897 approved on 28 December 2012). In the laboratory, specimens were acclimated for seven days at 17 °C with constant aeration in a photoperiod chamber with a 12 h light-dark cycle and fed twice a day with a 1:1 mixture of two cultures of nontoxic microalga species, *I. galbana* (3 × 10^6^ cells/L) and *T. suecica* (12 × 10^6^ cells/L). After acclimatization, mussels were randomly divided into two groups (n = 30 per experimental group) (Figure 7): a control group fed only with the microalga mixture used during acclimation period, and a treatment group additionally fed with 100,000 cells/L of the DSP toxin-producing alga *P. lima*. The culture of *P. lima* (strain AND-A0605) was obtained from the Quality Control Laboratory of Fishery Resources (Huelva, Spain). The treatment group was fed, four times a day, with 100,000 cells/L of *P. lima* during 48 h. These exposure characteristics were selected based on the results obtained in previous works by our research group in which these conditions showed the most interesting response at both the cytogenotoxic and the transcriptional level [10,12]. Cell concentrations of the nontoxic microalga cultures were determined by means of a Thoma cell counting chamber (Marienfeld, Lauda-Köningshofen, Germany), while that of the *P. lima* culture was estimated using the Sedgwich-Refter counting slide (Pyser-Sgi, Edenbridge, UK) after fixation with Lugol’s solution. After exposure, 12 individuals from each group—control and treatment—were dissected for digestive gland and gill tissues. These tissues were frozen in liquid nitrogen and stored at −80 °C until their use for RNA extraction, while the remaining individuals were used to estimate OA—the main DSP toxin—accumulation in the mussels by means of High Performance Liquid Chromatography/Mass Spectrometry (HPLC/MS). HPLC/MS analyses were carried out by the chromatography unit at Servizos de Apoio á Investigación (SAI)-University of A Coruña, following the protocol of the European Union Reference Laboratory for Marine Biotoxins [68].

### 5.2. RNA Extraction

Total RNA of digestive gland and gill from six control and six treated mussels was individually extracted using TRIzol (Invitrogen, Carlsbad, CA, USA), according to the manufacturer’s instructions (Figure 7). Isolated RNA was initially quantified using a NanoDrop 1000 spectrophotometer (Thermo Scientific, Waltham, MA, USA). With the aim of reducing inter-individual variability, these RNAs were pooled (in equal quantities) in groups of three to provide a template for Illumina libraries (Figure 7). Additionally, quantity and integrity of RNA pools were checked using a Qubit 2.0 fluorometer (Life Technologies, Saint-Aubin, France) and an Agilent 2100 Bioanalyzer (Agilent Technologies, Santa Clara, CA, USA), respectively.

### 5.3. Library Preparation and Sequencing

cDNA libraries were prepared and sequenced by Sistemas Genómicos (Valencia, Spain). Eight cDNA libraries were obtained from the digestive gland and the gill of mussels (two from control mussels and two from mussels exposed to *P. lima*, for each tissue, Figure 7). Poly(A)+mRNA fraction was isolated from total RNA and cDNA libraries were constructed following Illumina’s recommendations. cDNA libraries were sequenced using an Illumina HiSeq 2000 sequencer (Illumina, San Diego, CA, USA) and a paired-end sequencing strategy (100 × 2 bp). Raw data are accessible from the NCBI Short Read Archive (SRA accession: SRP158485).

### 5.4. De Novo Assembly

A preliminary bioinformatic analysis was performed by Sistemas Genómicos (Valencia, Spain). Initially, short sequence reads were quality checked using FastQC [69] and the TrueSeq adapters were trimmed using Trim Galore software version 0.3.3 (Babraham Bioinformatics, Cambridge, UK), keeping those reads with a mean phred score >30. With the aim of obtaining a reference transcriptome, all generated results were combined in a single data set. Then, low quality reads were re-identified and removed using PrinSeq-lite software version 0.20.4 [70], while duplicate reads were then removed using FastX-Toolkit (fastx_collapser option) [71]. Subsequently, de novo transcriptome assembly was conducted with the software Oases (version 2.0.9) and Trinity (version 2.1.1). Both assemblies were correlated by combining contigs with sequence similarity (>90% homology) using cd-hit (version 4.6). Potential ORFs were predicted using TransDecoder (version 2.0) with default settings. Then, each library was mapped against the reference transcriptome obtained in the previous step using Bowtie2 (version 2.2.6) and high quality reads were selected—high mapping quality with a 1 × 10^−4^ error probability—to increase count expression resolution. Finally, expression inference was carried out using the counts of properly paired reads by transcript.

### 5.5. Differential Expression, Functional Annotation and Functional Enrichment Analysis of DEGs

The expression of each sample was normalized by library size (initial number of reads) using the R package DESeq2 version 1.8.2 [72] (R software version 3.2.3 [73]) based on a negative binomial distribution, with the aim of analyzing differential expression. Those genes with a fold change lower than −2 or higher than 2, and an adjusted *p*-value < 0.05 were considered differentially expressed. Additionally, the method for controlling FDR was used to calculate the adjusted *p*-values [74].

DEGs were initially annotated using blastx against UniProt database and blastn against the NCBI nucleotide database, using an E-value threshold of 0.01. Subsequently, sequences annotated with RNAs were identified, while sequences associated with *P. lima* were removed from further analysis. Additionally, DEGs were re-annotated by a blastx analysis (ncbi-blast/2.3.0+)—using an e-value of 1 × 10^−6^ as cut-off—performed through the Supercomputing Centre of Galicia (CESGA). Subsequently, to know the biological processes, molecular functions and cellular components related to DEGs, annotated sequences were analyzed using GO implemented in Blast2GO software [75,76]. A functional enrichment analysis was performed using the Pfam [77] functional information, with the aim of annotating protein domains. Additionally, a subset of annotated DEGs was selected based on their biological function and their gene expression levels were represented in a heat map using CIMminer [78].

### 5.6. Real-Time Quantitative PCR Validation

A subset of annotated DEGs was selected based on their biological function to validate their gene expression using real-time qPCR. Reference genes for expression quantification were selected among six potential candidate housekeeping genes, including two primers for 18S ribosomal RNA (18S) [79], ribosomal protein S4 (rpS4), glyceraldehyde 3-phosphate-dehydrogenase (GAPDH) [40], elongation factor 1 (EF1) [10] and tropomyosin (TPM). TPM primers were designed as part of this work from an annotated gene with very stable expression levels. These primers and the specific primers to amplify the selected DEGs were designed using the Universal Probe Library software [80] (Roche Diagnostics, Mannheim, Germany). Primer specificities were verified using agarose gel electrophoresis, showing one single DNA product of the expected length. Two different algorithms, Normfinder and BestKeeper, were initially used to rank candidate reference genes according to their stability in the digestive gland and to decide on the optimal number of reference genes required for accurate normalization. Normfinder was used with R version 3.0.1 [73] and BestKeeper is an Excel-based tool that uses pairwise correlations [81]. Whenever BestKeeper analysis showed genes with SD values > 1, those genes were excluded from correlation coefficient calculations. Subsequently, results were checked using RefFinder [82], a web-based tool that integrates four different algorithms (Normfinder, BesKeeper, GeNorm and Delta Ct).

RNA samples from those individuals previously used for library preparation were used for the real-time qPCR validation. Four independent biological replicates and two technical replicates were analyzed together using the sample maximization approach [83]. cDNA was synthesized using 1 µg of RNA using the First Strand cDNA Synthesis kit according to the manufacturer’s instructions (Roche Diagnostics, Mannheim, Germany). qPCR amplifications were carried out using the FastStart Essential DNA Green Master kit (Roche Diagnostics, Mannheim, Germany) following the manufacturer’s instructions with the following modifications. All reactions were performed in a final volume of 20 µL of master mix containing 6.4 µL H_2_O, 0.8 µL of each primer (10 µM), 10 µL of the SYBR Green Mix (Roche Diagnostics, Mannheim, Germany) and 2 µL of each reverse transcribed RNA (cDNA). Reactions consisted of an initial denaturation step of 10 min at 95 °C, followed by an amplification of the target cDNA for 40 cycles (denaturation at 95 °C for 10 s, annealing at 60 °C for 10 s, elongation at 72 °C for 10 s), melting curve analysis (1 cycle at 95 °C for 5 s, 65 °C for 60 s and 95 °C for 1 s), and cooling at 40 °C for 20 s. Specificity of the qPCR product was analyzed by melting curve analysis.

Efficiency of the reaction for each mRNA was determined using LinRegPCR 2014.x software [84]. Gene relative expression levels were normalized using rpS4 and TPM as reference genes. For data analyses, Cq values were extracted with the qPCR instrument software LightCycler Software 1.5.0 (Roche Diagnostics, Mannheim, Germany). Cq values were then exported to Excel (Microsoft, Redmond, WA, USA), and differences in expression were calculated using the Pfaffl method with two reference genes [85]. Whenever a single individual sample showed a Cq value with an over five point difference to the mean Cq for the condition, that value was considered an amplification error, therefore, that sample was removed and analyses were carried out using three biological replicates instead of four. Normalized relative quantities (NRQ) for each gene were represented in bar plots (control vs. treatment) using GraphPad Prism version 6 (GraphPad Prism Software Inc., La Jolla, CA, USA). For better visualization of results some data were log transformed for graphic representation. Differences in gene expression between control and treatment samples were determined by Mann-Whitney non-parametric U test using the SPSS IBM software package version 22 (IBM, Armon, NY, USA). An additional analysis to confirm the obtained gene expression differences was conducted in REST 2009 (Qiagen, Hilden, Germany) [86].

## Figures and Tables

**Table 1:** Summary of reference transcriptome assembly for *M. galloprovincialis*.

**Total number of contigs**	95,702	**L25**	1682 bp
**Total length**	71,623.079 Kb	**N50**	21,152
**Maximum contig length**	16,082 Kb	**L50**	1062 bp
**Minimum contig length**	102 pb	**N75**	42,376
**Average contig length**	748 bp	**L75**	668 bp
**N25**	7537	**%GC**	33.20%

**Table 2:** List of the 25 putative top up-regulated genes (ordered by *p*-value) in response to early concentrations of DSP toxins in the digestive gland of *M. galloprovincialis*.

Sequence ID	Description	Length (bp)	baseMean	Log2FC	FC	*p*-Value	Adjusted *p*-Value
ci|000006456|Bact|Sample_MGT2|2	cytochrome c oxidase subunit 1, partial	910	23,389.22	7.09	136.29	1.96 × 10^112^	1.70 × 10^−107^
ci|000001182|Bact|Sample_MBT2|2	* ATP-synt_A	578	3975.18	7.21	147.76	6.65 × 10 ^−66^	1.92 × 10^−61^
Contig39610	ribosomal protein L23a, partial	1166	2205.27	7.92	243.02	3.89 × 10^−43^	6.71 × 10^−39^
ci|000005084|Bact|Sample_MGT2|2	cytochrome c oxidase subunit I	1848	12,611.71	6.23	75.08	1.91 × 10^−37^	2.75 × 10^−33^
Contig34888	NA	529	608.03	8.47	355.72	4.04 × 10^−35^	4.98 × 10^−31^
ci|000015505|Bact|Sample_MGT1|2	NA	588	1046.84	5.05	33.22	9.05 × 10^−35^	9.78 × 10^−31^
ci|000014133|Bact|Sample_MGT2|2	* Glyco_hydro_16	949	411.92	6.95	124.04	3.09 × 10^−34^	2.97 × 10^−30^
Contig22742	NA	1165	944.38	6.61	97.63	4.80 × 10^−33^	4.14 × 10^−29^
Contig33832	Kazal-like serine protease inhibitor domain-containing protein	507	514.55	6.70	103.84	6.63 × 10^−33^	5.20 × 10^−29^
ci|000004031|Bact|Sample_MGT1|2	* Porin_3	1024	888.45	5.19	36.44	3.14 × 10^−31^	2.09 × 10^−27^
ci|000016700|Bact|Sample_MGT1|2	† COX1_MYTED	750	12,157.03	6.86	116.52	2.32 × 10^−30^	1.43 × 10^−26^
ci|000022316|Bact|Sample_MBT1|2	* Ribosomal_L23	340	280.82	6.68	102.35	2.26 × 10^−28^	1.15 × 10^−24^
Contig17884	PREDICTED: 60 kDa SS-A/Ro ribonucleoprotein	1726	227.65	5.51	45.57	6.21 × 10^−25^	2.68 × 10^−21^
ci|000012420|Bact|Sample_MGT2|2	NA	560	761.81	9.97	1004.61	2.04 × 10^−24^	8.01 × 10^−21^
ci|000010593|Bact|Sample_MBC1|2	* Ribosomal_L7Ae	458	403.47	8.29	313.91	6.26 × 10^−23^	2.16 × 10^−19^
ci|000001186|Bact|Sample_MGT2|2	NA	316	209.87	6.26	76.55	7.11 × 10^−23^	2.36 × 10^−19^
ci|000001089|Bact|Sample_MGT2|2	NA	1106	224.57	5.35	40.77	1.03 × 10^−20^	3.08 × 10^−17^
Contig35276	NA	420	188.91	7.45	174.32	6.35 × 10^−20^	1.77 × 10^−16^
Contig38903	* Myticin-prepro	506	510.91	9.28	623.48	1.02 × 10^−19^	2.68 × 10^−16^
ci|000022507|Bact|Sample_MBT2|2	* Ribosomal_S9	1568	918.24	5.20	36.82	3.34 × 10^−19^	7.79 × 10^−16^
ci|000000480|Bact|Sample_MGT2|2	* Astacin	858	563.33	6.67	101.71	4.63 × 10^−19^	1.00 × 10^−15^
ci|000018470|Bact|Sample_MGT1|2	* Lectin_C	633	186.73	7.90	238.40	5.47 × 10^−19^	1.13 × 10^−15^
ci|000004710|Bact|Sample_MBC2|2	NA	1395	219.14	6.06	66.62	1.35 × 10^−18^	2.66 × 10^−15^
ci|000008308|Bact|Sample_MGT1|2	NA	464	637.37	5.04	32.92	1.82 × 10^−18^	3.49 × 10^−15^
ci|000004147|Bact|Sample_MGT2|2	NA	622	507.89	6.81	112.02	2.47 × 10^−18^	4.65 × 10^−15^

FC: Fold Change. NA: No gene annotation for the transcript. * Pfam result: protein containing the specified domain. † BlastUniProt result.

**Table 3:** List of the 25 putative top down-regulated genes (ordered by *p*-value) in response to early concentrations of DSP toxins in the digestive gland of *M. galloprovincialis*.

Sequence ID	Description	Length (bp)	baseMean	Log2FC	FC	*p*-Value	Adjusted *p*-Value
ci|000007816|Bact|Sample_MGC1|2	NA	539	7514.26	−7.48	−178.70	1.66 × 10^−82^	7.17 × 10^−78^
Contig22552	NA	622	131,251.75	−6.93	−121.52	5.49 × 10^−60^	1.18 × 10^−55^
Contig26868	NADH dehydrogenase subunit 5, partial	719	1916.17	−6.96	−124.62	2.16 × 10^−32^	1.56 × 10^−28^
Contig28135	40S ribosomal protein S10-like	559	406.63	−5.12	−34.81	5.46 × 10^−29^	3.14 × 10^−25^
Contig30578	* DUF1082	529	3132.77	−9.97	−1005.57	2.05 × 10^−28^	1.11 × 10^−24^
Contig28105	* SRCR	1419	329.84	−7.74	−213.17	2.60 × 10^−26^	1.25 × 10^−22^
ci|000000372|Bact|Sample_MGC1|2	NA	723	851.06	−6.15	−70.81	7.19 × 10^−26^	3.27 × 10^−22^
ci|000009048|Bact|Sample_MBC1|2	NA	703	359.37	−8.39	−334.64	1.14 × 10^−24^	4.67 × 10^−21^
Contig26906	NA	530	286.75	−8.12	−279.10	1.47 × 10^−23^	5.52 × 10^−20^
ci|000018684|Bact|Sample_MBC2|2	* Cytochrom_B_N_2	643	2708.10	−7.79	−221.90	5.93 × 10^−23^	2.13 × 10^−19^
ci|000000728|Bact|Sample_MGC2|2	NA	768	510.67	−9.66	−810.78	8.67 × 10^−23^	2.77 × 10^−19^
Contig29976	uncharacterized protein LOC567525 isoform X1/* Fibrinogen_C	1089	167.09	−6.83	−114.10	6.31 × 10^−21^	1.95 × 10^−17^
ci|000000734|Bact|Sample_MGC2|2	* Zona_pellucida	1185	255.85	−9.02	−518.06	4.16 × 10^−20^	1.20 × 10^−16^
Contig26843	NA	984	230.17	−4.23	−18.76	7.68 × 10^−20^	2.07 × 10^−16^
ci|000002253|Bact|Sample_MGC2|2	PREDICTED: GTPase IMAP family member 7/* AIG1	1188	255.05	−7.83	−228.11	1.33 × 10^−19^	3.37 × 10^−16^
ci|000003979|Bact|Sample_MGC1|2	NA	1136	233.95	−8.88	−471.60	2.94 ×10^−19^	7.25 × 10^−16^
ci|000021317|Bact|Sample_MBC1|2	NA	834	1818.89	−6.97	−125.28	3.04 × 10^−19^	7.29 × 10^−16^
ci|000008655|Bact|Sample_MGC2|2	NA	374	1501.18	−8.07	−267.93	4.48 × 10^−19^	1.00 × 10^−15^
ci|000004674|Bact|Sample_MGC2|2	Perlucin	660	174.13	−5.61	−48.70	4.57 × 10^−19^	1.00 × 10^−15^
ci|000023153|Bact|Sample_MBT1|2	* COX1	605	4253.61	−6.85	−115.03	5.47 × 10^−19^	1.13 × 10^−15^
ci|000001983|Bact|Sample_MBC2|2	* KOW	607	4924.53	−2.78	−6.88	8.86 × 10^−19^	1.78 × 10^−15^
ci|000005149|Bact|Sample_MGC1|2	* TIG	612	148.86	−6.00	−64.19	2.90 × 10^−17^	5.11 × 10^−14^
ci|000009215|Bact|Sample_MGC2|2	* Glyco_hydro_10	946	136.75	−7.11	−138.62	4.08 × 10^−17^	7.06 × 10^−14^
Contig28020	NA	570	166.31	−8.45	−350.56	4.96 × 10^−17^	8.24 × 10^−14^
ci|000015516|Bact|Sample_MBT2|2	* Ribosomal_L22	1338	1356.05	−4.92	−30.37	1.19 × 10^−16^	1.87 × 10^−13^

FC: Fold Change. NA: No gene annotation for the transcript. * Pfam result: protein containing the specified domain.

**Table 4:** List of the 25 putative top up-regulated genes (ordered by *p*-value) in response to early concentrations of DSP toxins in the gill of *M. galloprovincialis*.

Sequence ID	Description	Length (bp)	baseMean	Log2FC	FC	*p*-Value	Adjusted *p*-Value
ci|000029194|Bact|Sample_MBT1|2	* EF-hand_1 and 7	508	3570.44	9.76	868.30	7.36 × 10^−98^	6.48 × 10^−93^
ci|000006043|Bact|Sample_MBT1|2	NA	471	4432.18	6.05	66.47	4.32 × 10^−60^	1.90 × 10^−55^
ci|000001929|Bact|Sample_MBT2|2	NA	690	803.53	7.61	195.15	4.55 × 10^−49^	1.00 × 10^−44^
ci|000002899|Bact|Sample_MBT1|2	NA	779	1066.24	9.48	715.09	9.38 × 10^−40^	1.65 × 10^−35^
Contig35833	NA	944	520.96	8.45	350.51	6.42 × 10^−29^	6.28 × 10^−25^
ci|000022507|Bact|Sample_MBT2|2	NADH dehydrogenase subunit 6	1568	475.01	3.90	14.88	1.10 × 10^−28^	9.70 × 10^−25^
ci|000017597|Bact|Sample_MBT1|2	* Antistasin	795	266.99	7.13	140.36	1.22 × 10^−28^	9.79 × 10^−25^
ci|000007496|Bact|Sample_MBT2|2	NA	745	425.52	6.39	84.07	4.01 × 10^−28^	2.94 × 10^−24^
ci|000020755|Bact|Sample_MBT2|2	† NU4M_MYTED	1483	849.56	5.15	35.44	1.44 × 10^−26^	9.05 × 10^−23^
ci|000025759|Bact|Sample_MBT2|2	NA	2007	257.03	7.56	188.10	1.81 × 10^−26^	1.06 × 10^−22^
Contig39610	* Ribosomal_L23	1166	453.85	6.46	87.80	3.94 × 10^−26^	2.16 × 10^−22^
Contig15942	NA	482	916.44	5.84	57.29	6.24 × 10^−26^	3.23 × 10^−22^
ci|000001411|Bact|Sample_MGC2|2	* HSBP1	580	421.11	4.55	23.41	1.82 × 10^−25^	8.92 × 10^−22^
ci|000005084|Bact|Sample_MGT2|2	* COX1	1848	3923.98	3.98	15.75	3.81 × 10^−25^	1.77 × 10^−21^
ci|000003417|Bact|Sample_MBT2|2	* Phospholip_A2_1	562	203.18	6.08	67.55	1.10 × 10^−23^	4.05 × 10^−20^
Contig20144	NA	2258	205.66	3.81	14.01	4.48 × 10^−23^	1.58 × 10^−19^
ci|000019916|Bact|Sample_MBT1|2	NA	768	241.64	7.15	141.88	1.42 × 10^−22^	4.45 × 10^−19^
ci|000001302|Bact|Sample_MBT1|2	NA	675	281.74	6.61	97.65	1.36 × 10^−21^	4.00 × 10^−18^
ci|000018492|Bact|Sample_MBT1|2	NA	1552	159.56	5.50	45.19	1.59 × 10^−21^	4.36 × 10^−18^
Contig13066	Calcyphosin-like protein	2325	853.76	3.68	12.85	2.01 × 10^−21^	5.35 × 10^−18^
ci|000018122|Bact|Sample_MBT2|2	* HYR and TMEM154	3321	2042.07	4.34	20.27	2.30 × 10^−21^	5.95 ×10^−18^
Contig12937	† RS27L_HUMAN	2183	321.15	7.71	210.04	3.66 × 10^−21^	9.21 × 10^−18^
ci|000000451|Bact|Sample_MGT2|2	NA	655	587.43	2.92	7.57	1.42 × 10^−20^	3.38 × 10^−17^
ci|000003122|Bact|Sample_MBT2|2	NA	1513	215.45	7.54	185.87	4.85 × 10^−20^	1.12 × 10^−16^
Contig40138	NA	584	136.63	6.17	72.15	7.64 × 10^−20^	1.72 × 10^−16^

FC: Fold Change. NA: No gene annotation for the transcript. * Pfam result: protein containing the specified domain. † Blast UniProt result.

**Table 5:** List of the 25 putative top down-regulated genes (ordered by *p*-value) in response to early concentrations of DSP toxins in the gill of *M. galloprovincialis*.

Sequence ID	Description	Length (bp)	baseMean	Log2FC	FC	*p*-Value	Adjusted *p*-Value
ci|000007038|Bact|Sample_MBC2|2	low-density lipoprotein receptor-related protein 8 isoform X1	689	1321.99	−9.45	−700.32	1.06 × 10^−53^	3.11 × 10^−49^
Contig3681	NA	895	1561.88	−10.25	−1216.97	9.98 × 10^−37^	1.46 × 10^−32^
Contig11592	NA	798	652.29	−9.55	−752.01	4.28 × 10^−31^	5.38 × 10^−27^
Contig1183	NA	581	802.66	−4.76	−27.10	9.28 × 10^−30^	1.02 × 10^−25^
Contig8105	NA	1717	199.06	−6.43	−86.51	3.62 × 10^−27^	2.45 × 10^−23^
ci|000015242|Bact|Sample_MGT2|2	NA	663	429.13	−7.24	−151.36	4.36 × 10^−25^	1.92 × 10^−21^
ci|000005973|Bact|Sample_MBC1|2	NA	1860	257.90	−4.59	−24.07	5.34 × 10^−25^	2.24 × 10^−21^
Contig10936	* Oxidored_q1	2797	18,810.53	−1.67	−3.18	3.68 × 10^−24^	1.47 × 10^−20^
ci|000000312|Bact|Sample_MBC1|2	NA	972	208.97	−7.32	−159.95	4.63 × 10^−24^	1.77 × 10^−20^
Contig6277	NA	702	2009.78	−10.35	−1303.40	6.01 × 10^−23^	2.04 × 10^−19^
ci|000016192|Bact|Sample_MBC1|2	* Ldl_recept_a and PRKCSH-like	946	4008.01	−2.65	−6.26	9.71 × 10^−23^	3.17 × 10^−19^
Contig3876	Predicted protein	536	295.82	−8.43	−344.34	5.01 × 10^−22^	1.52 × 10^−18^
Contig4774	neurocalcin homolog	1267	339.45	−4.83	−28.39	1.49 × 10^−21^	4.22 × 10^−18^
ci|000000823|Bact|Sample_MBC2|2	NA	803	325.92	−3.99	−15.91	1.06 × 10^−20^	2.59 × 10^−17^
Contig6059	NA	486	5331.78	−1.93	−3.82	8.13 × 10^−20^	1.76 × 10^−16^
Contig7283	cytochrome c oxidase subunit I	2879	132.24	−5.09	−34.06	9.51 × 10^−20^	1.99 × 10^−16^
ci|000015433|Bact|Sample_MBC1|2	cytochrome c oxidase subunit I	1136	17,057.57	−3.52	−11.49	3.00 × 10^−19^	5.74 × 10^−16^
ci|000004320|Bact|Sample_MBC1|2	* Lipoxygenase	1950	564.59	−9.46	−703.80	3.48 × 10^−19^	6.24 × 10^−16^
ci|000001144|Bact|Sample_MBC2|2	NA	434	221.23	−8.61	−389.88	4.06 × 10^−19^	7.15 × 10^−16^
ci|000008127|Bact|Sample_MBC2|2	NA	2503	364.67	−10.03	−1043.76	7.20 × 10^−19^	1.22 × 10^−15^
ci|000005247|Bact|Sample_MBC1|2	NA	1246	153.48	−6.35	−81.80	1.26 × 10^−18^	2.09 × 10^−15^
ci|000001610|Bact|Sample_MBC1|2	NA	1057	341.22	−9.94	−982.30	1.93 × 10^−18^	3.11 × 10^−15^
ci|000000874|Bact|Sample_MBC2|2	NA	697	153.63	−5.02	−32.42	5.21 × 10^−18^	7.77 × 10^−15^
ci|000002263|Bact|Sample_MBC1|2	* Pfam-B_5682	1222	151.49	−4.77	−27.37	6.23 × 10^−18^	8.99 × 10^−15^
ci|000003990|Bact|Sample_MGT2|2	NA	521	310.73	−9.76	−866.01	2.46 × 10^−17^	3.23 × 10^−14^

FC: Fold Change. NA: No gene annotation for the transcript. * Pfam result: protein containing the specified domain.

**Table 6:** Pfam families significantly enriched (False Discovery Rate (FDR) adjusted *p*-value < 0.1) with seven or more differentially expressed genes in digestive gland.

Category	Number of Genes	*p*-Value
PF04548.11//AIG1	26	0.00248912
PF01926.18//MMR_HSR1	25	0.0029543
PF00059.16//Lectin_C	21	0.01366918
PF00100.18//Zona_pellucida	16	0.00403746
PF13499.1//EF-hand_7	14	0.00221868
PF13405.1//EF-hand_6	14	0.00355134
PF00036.27//EF-hand_1	13	0.00065889
PF13202.1//EF-hand_5	13	0.02872925
PF13833.1//EF-hand_8	12	0.02032022
PF00361.15//Oxidored_q1	10	0.00489835
PF00119.15//ATP-synt_A	8	0.00023995
PF10690.4//Myticin-prepro	8	0.02237525
PF07679.11//I-set	7	0.04744078

**Table 7:** Pfam families significantly enriched (FDR adjusted *p*-value < 0.1) with seven or more differentially expressed genes in gill.

Category	Number of Genes	*p*-Value
PF00386.16//C1q	36	5.2 × 10^−8^
PF00036.27//EF-hand_1	31	0.00035296
PF13499.1//EF-hand_7	29	0.00014495
PF13405.1//EF-hand_6	27	8.61 × 10^−5^
PF00147.13//Fibrinogen_C	25	0.01665835
PF13202.1//EF-hand_5	23	0.00079724
PF13833.1//EF-hand_8	20	0.00015502
PF10690.4//Myticin-prepro	13	0.01435613
PF00361.15//Oxidored_q1	13	0.03222834
PF07679.11//I-set	9	0.00010238
PF09458.5//H_lectin	9	0.00621282
PF01607.19//CBM_14	9	0.02592731
PB002965//Pfam-B_2965	9	0.03289021
PF13895.1//Ig_2	8	0.00039907
PF00119.15//ATP-synt_A	8	0.01065053
PF13927.1//Ig_3	7	0.00090571
PF00092.23//VWA	7	0.00272518
PF07686.12//V-set	7	0.01729404
PF03281.9//Mab-21	7	0.03056683

**Table 8:** Primers used in the real-time qPCR validation.

Gene Name	Abbreviation	Reference	E	Amplicon Size (bp)	Tm (°C)	Primers 5′→3′
Tropomyosin	TPM	ab000907.1	1.90	67	F-55.3 R-57.1	F-GATGCTGAAAATCGTGCAAC R-CGGTCTACTTCTTTTTGCAACTT
Ribosomal proteins S4	rpS4	Lozano et al. (2015)	1.83	138	F-58.8 R-60.3	F-TGGGTTATCGAGGGCGTAG R-TCCCTTAGTTTGTTGAGGACCTG
18S ribosomal RNA	18S	L33452.1	1.86	60	F-58.3 R-55.9	F-CCTGGAAAGGTCGGGTAAC R-AATTACAAGCCCCAATCCCTA
18S ribosomal RNA	18S-L33448	Cubero-Leon et al. (2012)	1.79	114	F-56.3 R-56.0	F-CATTAGTCAAGAACGAAAGTCAGAG R-GCCTGCCGAGTCATTGAAG
Glyceraldehyde 3-phosphate-dehydrogenase	GAPDH	Lozano et al. (2015)	1.92	114	F-59.4 R-58.4	F-AGGAATGGCCTTCAGGG R-TCAGATGCTGCTTTAATGGCTG
Elongation Factor 1	EF1	Suarez-Ulloa et al. (2013)	1.89	106	F-55.8 R-57.0	F-CCTCCCACCATCAAGACCTA R-GGCTGGAGCAAAGGTAACAA
Big defensin 2	BD2	Contig37896	1.83	110	F-60.3 R-59.3	F-TCTGAGCAGGGAGTATCAACAG R-TGGACAAAACAGCTACTAACAAGG
NADH dehidrogenase subunit 5	NADH5	Contig24266	1.86	90	F-53.7 R-56.5	F-GCAGTCATGCGCAAAAAG R-ACCCGGTACAAATATGGCTAAA
Dynactin-subunit-6-like	DYNA	Contig14551	1.89	60	F-58.9 R-58.9	F-AGTATTCTCAGGCATGGTTTCTG R-GGTTGTATAATTGGAGGCATGTG
GIY-YIG domain containing protein	GIY-YIG DC	ci|000000744|Bact|Sample_MBC1|2	1.83	70	F-57.6 R-55.3	F-AATCTACCAATTGCTTGTCTGTCA R-CGAAACGTAGTGTGCGAAAA
KAZAL domain containing protein	KAZAL DC	Contig33832	1.91	60	F-53.2 R-60.3	F-ATAATCGGCAGTGCAAAACA R-TTCCTTACTGAGTCAGTCG
Cytosolic phospholipase A-2 like	CPLA2	ci|000001655|Bact|Sample_MBT1|2	1.80	73	F-61.6 R-57.1	F-CCTGTACTGTGAGATTAGGTTATTGC R-CAGAAGGTTATTGACCGAAAGAA
Arachidonate 15-lipoxygenase B-like	ALOX15B	ci|000023941|Bact|Sample_MBT2|2	1.81	94	F-58.5 R-55.9	F-TGTTGTGAGTGAAGCAATAACTCTAA R-CGGAATAAATCG AGAGAACCA
Alpha-L-fucosidase-like	FUCA	ci|000010451|Bact|Sample_MBT1|2	1.87	74	F-61.0 R-55.3	F-GGAATTCCAGTAGGAATCAGTAGC R-TGGTAAATGCATACAAACCTGAA
H_Lectin domain containing protein	H_Lectin DC	Contig19341	1.85	73	F-56.5 R-55.3	F-CCCTTCTTTGCTTTAGATGCTT R-TTGATGGCCAGATTACGACA
Fibrinogen_C domain containing protein	Fibrinogen_C DC	ci|000024772|Bact|Sample_MBC1|2	1.86	67	F-57.3 R-59.4	F-AAGGTTGTCTCCAGCGTTTC R-CGGTGATGCCTCTACCAACT

E: primer efficiency; F: forward; R: reverse.

**Table 9:** Rank of six candidate reference genes for real-time qPCR calculated by Normfinder and BestKeeper analyses.

Rank	Normfinder	Stability	BestKeeper	SD	*r*
1	rpS4	0.07	rpS4	0.46	0.732
2	TPM	0.17	TPM	0.50	0.448
3	GAPDH	0.20	GAPDH	0.64	0.669
4	18S	0.37	18S	0.71	0.827
5	18S-L33448	0.76	18S-L33448	1.08	
6	EF1	1.78	EF1	2.91	

SD: standard deviation; *r*: coefficient of correlation between each gene and the BestKeeper index.

## Supplementary Materials

The following are available online at http://www.mdpi.com/2072-6651/10/10/417/s1, File S1: Nucleotide sequences—in fasta format—of all differentially expressed genes (DEGs), File S2: List of DEGs. Each spreadsheet shows DEGs from the comparison of either the same tissue under different conditions—MBT_vs_MBC_DEGs for gills and MGT_vs_MGC_DEGs for digestive gland—or two tissues under the same condition—MGT_vs_MBT_DEGs for treated digestive gland and gill. For each DEG, sequence ID, baseMean, length, Log2 Fold Change (FC), FC, *p*-value and adjusted *p*-value are given. Also, Blast_nucleotide, Blast_UniProt and Pfam columns show the best hit against Nucleotide, UniProt and Pfam databases, respectively, File S3: List of Pfam families functionally enriched. Each spreadsheet shows DEGs from the comparison of the same tissue under different conditions—MGT_vs_MGC for digestive gland and MBT_vs_MBC for gill. For each Pfam category, number of genes, *p*-value, expression patterns and gene IDs are given.
